# Physical activity reduces risk for colon polyps in a multiethnic colorectal cancer screening population

**DOI:** 10.1186/1756-0500-5-312

**Published:** 2012-06-20

**Authors:** Nelson F Sanchez, Bryan Stierman, Said Saab, Divya Mahajan, Howa Yeung, Fritz Francois

**Affiliations:** 1 Memorial Sloan Kettering Cancer Center, New York, NY, USA; 2 NYU Langone Medical Center, New York, NY, USA; 3 Msc, NYU Langone Medical Center, New York, NY, USA

**Keywords:** Physical activity, Colorectal polyps, BMI

## Abstract

**Background:**

Identifying modifiable factors that influence the epidemiology of colorectal cancer incidence among multiethnic groups might be informative for the development of public health strategies targeting the disease. Minimal data exists describing the impact of physical activity on colorectal polyp risk in United States minority populations. The aim of this study is to evaluate the relationship of exercise on the prevalence of polyps in a multiethnic colorectal cancer screening population.

**Results:**

We enrolled 982 patients: 558 Hispanic, 202 Asian,149 Black, and 69 White. Patients who reported exercising one or more hours weekly had a lower prevalence of any polyps (25.3% vs 33.2%, *P* = 0.008) as well as adenomas (13.8 vs. 18.9%, *P* = 0.03) compared to those who did not exercise. Black and Hispanic patients and those who were overweight or obese also had lower prevalence of polyps if they led an active lifestyle. Multivariate analysis revealed that age >55, male sex, and Black race/ethnicity were positively associated with the presence of adenomas, while a history of exercising one hour or more weekly was an independent negative predictor for the presence of adenomas anywhere in the colon (OR 0.67; 95% CI 0.4 - 0.9, *P* = 0.03).

**Conclusions:**

Exercising one hour per week was associated with a lower prevalence of polyps and adenomas when compared to those who exercised less or not at all. An active lifestyle provides benefits to groups who are at risk for colorectal cancer, such as Blacks. It also provides significant protection to overweight and obese individuals. Public health initiatives should promote physical activity as a cancer prevention tool in multiethnic populations.

**Trial registration:**

none

## Background

As the second leading cause of overall cancer death in the United States, colorectal cancer (CRC) remains an important preventable public health concern. In 2010 an estimated 142,570 new cases of CRC were diagnosed and there were 51,370 associated deaths [1]. There are notable racial/ethnic differences in the epidemiology of the disease. The National Cancer Institute’s 2007 Surveillance Epidemiology and End Results (SEER) Report determined a colorectal cancer incidence rate of 54.71/100,000 for Blacks versus 43.16 for Whites, 39.78 for Asians, and 35.84 for Hispanics [2]. Identifying modifiable factors that influence the epidemiology of colorectal cancer incidence among multiethnic groups might be informative for the development of public health strategies targeting the disease.

Physical activity has been estimated to reduce colon cancer risk by 40-50% for individuals who engage in at least moderate levels of exercise (e.g.: brisk walking for 3–4 hours/week) [3,4]. The protective role of physical activity may also extend to the development of pre-cancerous colorectal polyps [5-14]. Current data suggest that the benefit of physical activity is greatest for large or severely dysplastic polyps [11-14]. The mechanism of this benefit is not known, but it may involve reductions in insulin levels, systemic inflammation, and abdominal adiposity [15,16].

To date, there is minimal data describing the impact of exercise on colorectal polyp risk in minority populations. Published research describing the benefits of physical activity among U.S. minorities has been limited to Black populations [17,18], and none have described the impact of exercise on colorectal polyp risk in a study sample that includes Hispanics, Asians, Blacks, and Whites. Furthermore, few studies have examined the impact of physical activity on polyp distribution, particularly in the proximal colon where CRC is more likely in Blacks compared to Whites and other racial/ethnic groups [19-21]. We hypothesized that physical activity reduces the risk of polyps throughout the colon, regardless of ethnic background. This prospective study aims to evaluate the association between physical activity and risk of colorectal pathology in an urban, multiethnic population presenting for screening colonoscopy.

## Methods

Study population. Patients presenting for a screening colonoscopy at Bellevue Hospital were approached for enrollment. Bellevue is the largest public hospital in New York City and serves a diverse population that reflects the city’s multiethnic composition. Patients range from American citizens who have lived in New York City for several decades to undocumented residents who are less familiar with the regional lifestyle and cultural behaviors. The patients are predominantly Medicaid beneficiaries and come from low-income households.

Between January 2002 and December 2010, consecutive patients presenting for colonoscopy were approached to voluntarily participate in this study. Patients were included in the statistical analysis if they were asymptomatic average-risk adults at least 45 years of age. Asymptomatic was defined as the absence of gastrointestinal symptoms, including abdominal pain, constipation, diarrhea, change in stool caliber or frequency, unexplained weight loss, rectal bleeding, or other alarm symptoms suggestive of an underlying malignancy. Average risk was defined as no personal history of colorectal cancer, adenomatous polyps, or inflammatory bowel disease, and no family history of colorectal neoplasia in a first-degree relative [22-24].

Patients were subsequently excluded from the statistical analysis if they were HIV positive, had a positive fecal occult blood test, a flexible sigmoidoscopy or barium enema within the last 5 years, or if they had a colonoscopy within the last 10 years. Patients were excluded from further analyses beyond baseline if their colonoscopy was not complete to the cecum or if their bowel preparation was assessed as poor. The study protocol was reviewed and approved by the Institutional Review Board at NYU Medical Center.

Study design. A trained research assistant using a standardized scripted questionnaire obtained demographic and clinical information on all enrolled patients prior to colonoscopy. For non-English participants, the interview was conducted with the assistance of “TEMIS”, Team/Technology Enhanced Medical Interpreting System, a simultaneous translation service that has been implemented successfully at Bellevue Hospital Center. Height and weight were measured using the same column scale with a telescoping height rod while patients were barefooted and wearing light clothing. Information collected on each patient included age, gender, race/ethnicity, place of birth, and usage of dietary supplements (eg: folic acid, fiber, and vitamin supplements). Patients self-reported racial/ethnic designations as Black, Asian, White, and Hispanic. We assessed physical activity with three specific questions: 1) Do you regularly exercise? 2) How many hours of exercise per week?, and 3) How many years of exercise?

Prior to colonoscopy, patients received a bowel preparation that consisted of an oral sodium phosphate solution or a polyethylene glycol-based electrolyte solution. Trained endoscopists performed evaluation of the colon to the cecum after administration of intravenous meperidine and midazolam for conscious sedation. During the exam, the location and size of each polyp was recorded. The size of small polyps was estimated with the use of an open-biopsy forceps, which is 7 mm in diameter. A dedicated gastrointestinal pathologist reviewed all retrieved polyps and biopsies of any masses or other abnormalities. Advanced adenomas were defined as adenomas ≥ 10 mm in diameter or any adenoma, regardless of size, with villous histology, high-grade dysplasia, or adenocarcinoma [22].

Study outcomes. The primary outcome evaluated was the relationship between physical activity and the prevalence of colon polyps. We sought to compare individuals who exercised for at least one hour per week (defined as active) with those who exercised less frequently or not at all (defined as sedentary). The impact of exercise as defined by one hour of physical activity per week has been demonstrated to have cardiovascular and metabolic health benefits [25]. As such, we analyzed the impact of a comparable timeframe on adenoma prevalence.

Statistical analysis. Continuous variables were compared using the unpaired 2-tailed *t*-test or Mann–Whitney *U* test. Data are expressed as mean ±SD for those variables that were normally distributed, and as medians and interquartile range (25th to 75th percentiles) for those with a non-normal distribution. Categorical variables were compared using the Chi-square test or Fisher’s exact test (for sample sizes of n < 5).

Multivariable logistic regression analysis was used to assess the effect of ethnicity and other factors on the odds of having a neoplastic lesion detected by colonoscopy. The strength of the associations between these variables and the presence of colonic polyps are expressed as odds ratios (OR) with 95% confidence intervals (CI). With an alpha of 5%, the study was powered at 80% to detect at least a 5% difference in adenoma prevalence according to exercise history. Statistical analysis was performed using SPSS software PASW Statistics GradPack 18 for Windows (SPSS Inc., Chicago, IL), and a two-tailed p-value of < 0.05 was considered statistically significant.

## Results

Patient demographics and clinical characteristics. A total of 1,862 patients were enrolled in the study, and 982 of them met the eligibility criteria for inclusion in the analysis. A total of 548 patients reported an active lifestyle with at least one hour of exercise weekly (55.8%) while 434 (44.2%) reported less or no exercise (sedentary). We selected a threshold of one hour of weekly exercise as this was the point in our sample where we noted significant differences in patients’ polyp and adenoma prevalence. The two comparison groups did not differ significantly in gender, age, BMI, aspirin use, alcohol use, tobacco use, red meat consumption, and folate or fiber supplementation (Table 1). Active patients were less likely to be hypertensive than their sedentary counterparts (46.1 vs 52.3%, *P* = 0.052). Also, active patients were more likely to take multivitamin supplements than their sedentary counterparts (42.5 vs 36.4%, *P* < 0.01)

**Table 1:** Baseline demographic characteristics of the 982 evaluated individuals according to exercise history stratification (sedentary vs. active)

**Characteristic**	**Sedentary**	**Active**	**P value**
	**N = 434**	**N = 548**	
Male, n (%)	184 (42.4)	226 (41.2)	0.721‡
Mean age (yrs) ± SD	58 ± 7	58 ± 7	0.932*
BMI (kg/m^2^) ± SD	27.4 ± 5.4	27.1 ± 4.7	0.370*
Race/ethnicity, n (%)			
White, non-Hispanic	27 (6.2)	42 (7.7)	0.018‡
Black, non-Hispanic	60 (13.8)	89 (16.2)	
Hispanic	235 (54.1)	323 (58.9)	
Asian	109 (25.1)	93 (17.0)	
Hypertension, n (%)	226 (52.3)	252 (46.1)	0.052‡
Folate supplementation, n (%)	17 (3.9)	18 (3.3)	0.798‡
Fiber supplementation, n (%)	11 (2.5)	23 (4.2)	0.093‡
Multivitamin supplementation, n(%)	158 (36.4)	233 (42.5)	0.005‡
Aspirin, n (%) (current daily use)	116 (26.7)	146 (26.6)	0.459‡
Red meat consumption, n (%) (one or more servings/week)	357 (82.3)	416 (75.9)	0.741‡
Current Alcohol, n (%) (Any current use)	100 (23.0)	138 (25.2)	0.229‡
Current Tobacco, n (%)	53 (12.2)	53 (9.7)	0.352‡

*T-test.

‡Chi-Square Analysis.

Endoscopy findings. Colonoscopy was complete to the cecum in 100% of the 982 subjects included for analyses. A total of 281 subjects (28.6%) had at least one polypoid lesion observed on colonoscopy. The prevalence of adenomas was 15.8%, and the prevalence of advanced adenomas was 4.3%. While the majority of individuals with polyps (45.8%) had lesions in the distal colon alone, 33.7% had proximal lesions, and 20.5% had polyps in both the left and right colon.

Colonoscopy findings according to race/ethnicity. Blacks had the highest detection rate for any polyp (30.8%), adenoma (20.8%), and advanced adenoma (6.7%). White patients were significantly less likely to have any polyps detected on colonoscopy compared to other racial/ethnic groups (17.4 vs. 30.4%, *P* = 0.020). Pre-cancerous adenomatous polyps were significantly more prevalent in Black patients, compared to other groups (23.0 vs 15.3%, *P* = 0.020). On the other end of the risk spectrum, Asian patients were far less likely to have advanced adenomas compared to other racial/ethnic groups (1.0 vs 5.3%, *P* = 0.008). There was no significant racial/ethnic difference in the distribution of polyps in the left versus the right side of the colon.

Exercise history among the study subjects. Among the 548 patients who reported at least one hour of exercise weekly, the median number of years of exercise was 5.0 years (interquartile range 2.0-10.0). Asians were less likely than their non-Asian counterparts to report regular exercise (46.0 vs 58.0%, *P* = 0.002). Patients born outside the mainland United States were also less likely to report regular exercise than subjects born on the mainland (54.3 vs 66.1%, *P* = 0.01). Although 88% of our subjects were born outside of the mainland United States, the majority of the study group (81.9%) had lived in the United States for more than ten years. Consistent with this finding no significant difference was found in the main outcome of polyp prevalence based on immigrant status alone. Patients born in the United States were as likely to have polyps as patients born outside the United States (29.6 vs 29.4%, *P* = 0.974)

**Figure 1:**
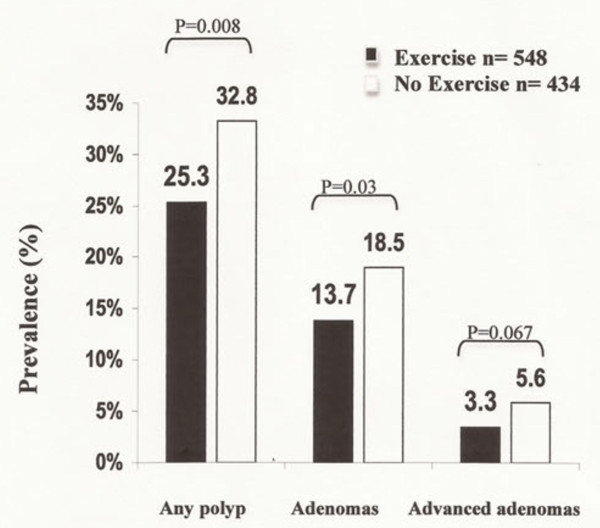
**Colonic findings according to exercise history.** Prevalence of any polyps, adenomas, and advanced adenomas among 548 patients who reported at least one hour of weekly exercise and 434 patients who reported no regular exercise. Variables analyzed with the Chi-square test.

Effect of exercise on polyp prevalence. Patients who reported at least one hour of exercise weekly (active lifestyle) were less likely to have any polyps on colonoscopy compared to those who did not regularly exercise (25.2 vs 33.2%, *P* < 0.01; Figure 1). Consistent with this finding, patients with active lifestyles were also less likely to have an adenoma on colonoscopy compared to their counterparts with more sedentary lifestyles (13.8 vs 18.9%, *P* = 0.03). Similarly, individuals who exercised were less likely to have advanced adenomas than those who did not exercise; however, this difference did not reach statistical significance (3.4 vs 5.8%, *P* = 0.06).

Among individuals who reported an active lifestyle, exercising three or more hours per week, trended towards a lower prevalence of adenomas, compared to those who exercised two hours or less (11.4 vs. 15.4%, *P* = 0.094). This protective trend extended to advanced adenomas (3.3 vs 5.4%, *P* = 0.116). Consistent with this finding individuals with a history of exercising five years or more trended towards fewer advanced adenomas, compared to those with a more recent exercise history (2.4 vs. 3.4%, *P* = 0.138).

The benefits of physical activity extended to all areas of the colon (Table 2). Active subjects had a lower prevalence of any polyps, adenomas, and advanced adenomas, for each location assessed; however, this did not always reach statistical significance. Active patients were less likely to have adenomas in the distal colon than sedentary patients (4.6 vs 8.1%, *P* = 0.043). We also observed a protective trend in the proximal colon among active patients, but these results did not reach statistical significance.

**Table 2:** Comparison of type and location of colonoscopy findings according to exercise history

	**Sedentary**	**Active**	**P value**
	**N = 434**	**N = 548**	
Any polyp			
Distal, n (%)	67 (15.4)	60 (10.9)	0.134
Proximal, n (%)	47 (17.2)	43 (7.8)	0.201
Distal & proximal, n (%)	30 (10.9)	27 (4.9)	0.424
Adenomas			
Distal, n (%)	35 (8.1)	25 (4.6)	0.043
Proximal, n (%)	35 (8.1)	35 (6.4)	0.457
Distal & proximal, n (%)	30 (6.9)	27 (4.9)	0.424
Advanced adenomas			
Distal, n (%)	13 (3.0)	10 (1.8)	0.295
Proximal, n (%)	8 (1.8)	7 (1.3)	0.554
Distal & proximal, n (%)	4 (0.9)	0	0.029

*Note*. Categorical variables were analyzed with Chi-Square test or the Fisher exact test (for sample sizes of n < 5 responses).

Effect of exercise on polyps according to race/ethnicity. Blacks and Hispanics benefited the most from physical activity (Table 3). Hispanics who exercised one hour or more per week were less likely to have adenomas than Hispanics who did not exercise regularly (11.2 vs 21.4%, *P* < 0.01). For Black patients with an active lifestyle, the prevalence of advanced adenomas was significantly less compared to Black patients with a sedentary lifestyle (2.4 vs. 13.6%, *P* = 0.01). White and Asian subjects did not benefit substantially from physical activity in this cohort.

**Table 3:** Analysis of Exercise and Polyp Prevalence with Ethnicity Subsets

**Colonoscopy Finding**	**Sedentary**	**Active**	**P value**
	**N = 434**	**N = 548**	
Any polyp			
*Ethnicity*			
Hispanic, n (%)	76 (17.5)	77 (14.1)	0.059
Black, n (%)	23 (5.3)	23 (4.2)	0.165
Asian, n (%)	35 (8.1)	22 (4.0)	0.188
White, n (%)	4 (0.9)	6 (1.1)	0.772
Any adenoma			
*Ethnicity*			
Hispanic, n (%)	48 (11.1)	34 (6.2)	0.002
Black, n (%)	14 (3.2)	17 (3.1)	0.618
Asian, n (%)	14 (3.2)	16 (2.9)	0.323
White, n (%)	2 (0.5)	3 (0.5)	0.942
Advanced adenoma			
*Ethnicity*			
Hispanic, n (%)	13 (3.0)	12 (2.2)	0.295
Black, n (%)	8 (1.8)	2 (0.4)	0.010
Asian, n (%)	0	2 (0.4)	0.116
White, n (%)	2 (0.5)	1 (0.2)	0.366

*Note*. Categorical variables were analyzed with Chi-Square test or the Fisher exact test (for sample sizes of n < 5 responses).

Effect of exercise on polyps according BMI. Since the prevalence of any polyps was greatest among individuals with an abnormal BMI compared to their normal BMI counterparts (30.4 vs. 27.5%, *P* = 0.343) we next evaluated the effect of exercise on polyp prevalence in this subgroup. Overweight and obese subjects who exercised were significantly less likely to have any adenomas (13.5 vs. 20.5%, *P* = 0.023) or advanced adenomas (2.5 vs. 6.3%, *P* = 0.011), compared to their counterparts who did not exercise. This trend persisted among overweight and obese Hispanics (Table 4). Overweight and obese Hispanics who exercised were significantly less likely to have adenomas compared to their counterparts who did not exercise (9.4 vs 21.2%, *P* < 0.01). Active overweight and obese Blacks were less likely to have advanced adenomas compared to their counterparts who did not exercise (0.2 vs 1.4%, *P* = 0.021). We did not observe these differences among Whites and Asians.

**Table 4:** Analysis of Exercise and Polyp Prevalence with BMI and Ethnicity subsets

**Colonoscopy Finding**	**Sedentary**	**Active**	**P value**
	**N = 434**	**N = 548**	
Any polyp			
*BMI <25*			
Hispanic, n (%)	24 (5.5)	22 (4.0)	0.144
Black, n (%)	6 (1.4)	6 (1.1)	0.434
Asian, n (%)	16 (3.7)	11 (2.0)	0.465
White, n (%)	2 (0.5)	1 (0.2)	0.476
*BMI* ≥ 25			
Hispanic, n (%)	52 (12.0)	55 (10.0)	0.275
Black, n (%)	17 (3.9)	17 (3.1)	0.196
Asian, n (%)	19 (4.4)	11 (2.0)	0.239
White, n (%)	2 (0.5)	5 (0.9)	0.551
Any adenoma			
*BMI <25*			
Hispanic, n (%)	13 (3.0)	14 (2.6)	0.424
Black, n (%)	3 (0.7)	4 (0.7)	0.826
Asian, n (%)	8 (1.8)	7 (1.3)	0.988
White, n (%)	0	1 (0.2)	0.329
*BMI* ≥ 25			
Hispanic, n (%)	35 (8.1)	20 (3.6)	0.003
Black, n (%)	11 (2.5)	13 (2.4)	0.432
Asian, n (%)	6 (1.4)	9 (1.6)	0.146
White, n (%)	2 (0.5)	2 (0.4)	0.624
Advanced adenoma			
*BMI <25*			
Hispanic, n (%)	6 (1.4)	6 (1.1)	0.622
Black, n (%)	2 (0.5)	1 (0.2)	0.312
Asian, n (%)	0	1 (0.2)	0.289
White, n (%)	0	1 (0.2)	0.329
*BMI* ≥ 25			
Hispanic, n (%)	7 (1.6)	6 (1.1)	0.351
Black, n (%)	6 (1.4)	1 (0.2)	0.021
Asian, n (%)	0	1 (0.2)	0.242
White, n (%)	2 (0.5)	0	0.068

*Note*. Categorical variables were analyzed with Chi-Square test or the Fisher exact test (for sample sizes of n < 5 responses).

Predictors of colonic neoplasia. Both univariate and multivariable logistic regression analyses were performed in order to determine factors that were associated with colonic adenomas (Table 5). In the univariate analysis, age, ethnicity, and exercise were significantly associated with colonic adenomas. In the multivariable analysis, age >55, male sex, and Black race/ethnicity remained positively associated with the presence of adenomas, while a history of exercising one hour or more per week was an independent negative predictor for the presence of adenomas anywhere in the colon (OR 0.67; 95% CI 0.4 - 0.9, *P* = 0.03).

**Table 5:** Relative odds ratio of colonic adenomas in 982 study subjects

	**Unadjusted**		Adjusted^*^	
	**OR [95% CI]**	***p*Value**	**OR [95% CI]**	***p*Value**
Age (yr)				
45–54, n = 359	1.0 [reference]	--	[reference]	--
55–64, n = 451	1.7 [1.1-2.5]	0.014	1.6 [1.1-2.5]	0.026
65–74, n = 156	2.8 [1.7-4.6]	0.001	3.1 [1.8-5.3]	0.001
75 and older, n = 16	8.2 [2.9-23.0]	0.001	9.2 [3.1-27.4]	0.001
Gender				
Female, n = 572	1.0 [reference]	--	[reference]	--
Male, n = 410	1.4 [0.97-1.93]	0.069	1.7 [1.2-2.4]	0.006
Ethnicity				
White, n = 69	1.0 [reference]	--	1.0 [reference]	--
Black, n = 149	2.5 [0.9-6.2]	0.061	3.0 [1.1-8.3]	0.03
Hispanic, n = 558	3.8 [1.4-10.2]	0.008	2.0 [0.7-5.3]	0.15
Asian, n = 202	2.4 [0.88-6.3]	0.088	2.1 [0.8-5.8]	0.15
BMI				
<25, n = 351	1.0 [reference]	--	1.0 [reference]	--
>25, n = 631	2.0 [0.3-12.3]	0.46	1.1 [0.8-1.7]	0.55
Exercise				
Sedentary, n = 434	1.0 [reference]	--	1.0 [reference]	--
Active, n = 548	0.68 [0.4-0.97]	0.03	0.67 [0.4-0.9]	0.03

*The values were derived from multivariable logistic regression after adjusting for all variables in the model (Age, Gender, Ethnicity, BMI, Exercise).

Folate supplementation, fiber intake and dietary vitamin supplementation were not significantly associated with a reduced risk of colorectal adenomas in this cohort.

## Discussion

In this multiethnic average-risk study population, an active lifestyle history, as reflected by exercising one hour or more per week, was associated with a significantly lower risk of colon polyps and adenomas. We observed the expected inverse trend between polyp prevalence and weekly hours of exercise. This association is consistent with other studies that have examined the protective properties of physical activity and colon polyps, where even low levels of physical activity have been associated with protective benefits [9,10,12,14,26,27]. Published data on the types of exercise performed is minimal, although some studies cite walking as a common form of exercise. In an observational nested study, walking at speeds > 3 mph was reported to be protective against advanced adenomas for men [27]. Currently, the U.S. Centers for Disease Control recommends that adults perform at least 150 minutes of moderate-intensity aerobic activity weekly or 75 minutes of vigorous-intensity aerobic activity weekly. This public health recommendation appears feasible for our patient population as over half currently report at least one hour of weekly exercise.

An important finding from this study is the observed benefit of exercise on polyp prevalence among overweight and obese subjects. Prior studies have reported an association between high body mass and an increased risk for colon polyps and colon cancer [28-31]. Efforts to combat this increased risk have focused on ways to reduce excess weight, namely, diet and exercise. Regular physical activity alone may confer benefits against important outcomes such as cancer risk reduction even if weight loss is not achieved. Exercise may mitigate the cancer-promoting effects of high body mass by through effects on hyperinsulinemia, systemic inflammation, and abdominal adiposity [9,10]. In addition, exercise may increase colon transit time and limit the interaction of the colonic mucosa with mutagenic intracolonic materials [32,33]. Our finding carries significant public health implications since it suggests that even light physical activity can provide cancer protection among average-risk subjects with increased BMI.

In the United States, colorectal cancer disproportionately affects Blacks, followed by Whites, Asians, and Hispanics [2]. These statistics were reflected in our sample, as we observed that Blacks had the greatest incidence of all polyps, adenomas, and advanced adenomas, underscoring the public health threat posed to this at-risk population. Notably, we found that routine exercise provides significant protection against colonic lesions among Blacks and Hispanics. This trend persisted among overweight and obese Hispanics and Blacks. These findings highlight the need for public health initiatives to promote physical exercise among low-income Black and Hispanic populations as cancer prevention tools.

Over half of our sample’s subjects identified as Hispanic. They reported descent from a diverse range of Latin American nations, most commonly Mexico, the Dominican Republic, and Ecuador. This makes our study unique in its robust representation of an infrequently studied population cohort. Although nationally Hispanics remain low-risk in terms of colorectal cancer incidence, these statistics may be biased by Hispanic immigrants that have not adopted American lifestyle and eating behaviors. As more Hispanics adopt American behaviors, colorectal cancer and polyp prevalence may approach those of White or even Black populations, depending on socioeconomic achievement. This lower risk population may have led to an overall low-end adenoma detection rate in our sample. We anticipated an adenoma detection rate in the 15-25% range [34]. Our sample’s overall adenoma detection rate was 15.8%, and the Hispanic subset’s detection rate was 14.7%. Our Hispanic sample reported similar physical activity levels as Blacks and Whites, making bias in our subsequent analyses minimal.

Surprisingly, we did not find exercise to have a significant benefit among Asians. This may be because a disproportionate number of Asian participants did not report any regular exercise. Furthermore Asians were less likely than their non-Asian peers to be obese and overweight, which may have potentially muted the beneficial impact of exercise. Future studies among Asian populations should explore the relationship between exercise and colon polyps. The benefits of exercise were also not observed among Whites. This is possibly due to the small sample size of White subjects in our study.

A limitation of this study is the absence of specific data on exercise type and level of intensity, which makes it difficult to make a specific recommendation on how people should exercise to reduce their risk for colon cancer. We can say that regular weekly exercise for at least one hour confers increased protection against colorectal polyps and adenomas. Another limitation is possible misinformation obtained from patients through translators. The term “exercise” may not translate well in specific languages and may explain why Asians disproportionately reported no exercise. The majority of our Asian subjects were of Chinese descent, speaking predominantly Mandarin or Cantonese dialects. We used medical translators provided by our medical institution, so this bias should be minimal.

## Conclusions

Our study indicates that an active lifestyle, as defined by at least one hour of exercise per week, is associated with a lower risk of colonic neoplasia. Exercise provides benefits among at-risk groups such as Blacks, and it also provides significant protection to overweight and obese people. To our knowledge, this is the first study to observe the protective nature of exercise on colorectal neoplasia within a multiethnic cohort. Further evaluation is warranted to define the molecular aspects of physical exercise on colorectal oncogenesis. Public health initiatives should incorporate promotion of physical activity as a cancer prevention tool in multiethnic ethnic groups.

## Competing interests

The authors declare that they have no competing interests.

## Authors’ contributions

NFS participated in patient recruitment, statistical analysis, and manuscript preparation. BS participated in patient recruitment, data entry and analysis as well as manuscript preparation. SS participated in patient recruitment and manuscript preparation. DM participated in patient recruitment, data entry, and manuscript preparation. HY participated in patient recruitment and manuscript preparation. FF participated in the design of the study, patient recruitment, sample procurement, statistical analysis, and manuscript preparation. All authors read and approved the final manuscript.

## Disclosure

None of the authors have competing interest to declare in relation to this manuscript.

## References

[B1] Surveillance Epidemiology and End Results. (2008). Colorectal Cancer Facts and Figures 2008 http://www.cancer.org/Research/CancerFactsFigures/colorectal-cancer-facts--figures-2008-2010

[B2] Howlader N Noone AM Krapcho M SEER Cancer Statistics Review, 1975–2008 2011 National Cancer Institute, Bethesda, MD http://seer.cancer.gov/csr/1975_2009_pops09/, based on November 2010 SEER data submission, posted to the SEER web site, 2011

[B3] Colbert LH Hartman TJ Malila N Physical activity in relation to cancer of the colon and rectum in a cohort of male smokers Cancer Epidemiol Biomarkers Prev 2001 10 3 265 268 11303597

[B4] Colditz GA Cannuscio CC Frazier AL Physical activity and reduced risk of colon cancer: implications for prevention Cancer Causes Control 1997 8 4 649 667 10.1023/A:1018458700185 9242482

[B5] Wolin KY Yan Y Colditz GA Physical activity and risk of colon adenoma: a meta-analysis Br J Cancer 2011 104 5 882 885 10.1038/sj.bjc.6606045 21304525 PMC3048199

[B6] Friedenrich CM Neilson HK Lynch BM State of the epidemiological evidence on physcial activty and cancer prevention Eur J Cancer 2010 46 14 2593 2604 10.1016/j.ejca.2010.07.028 20843488

[B7] Spence RR Heesch KC Brown WJ Systematic review of the association between physical activty and colorectal cancer risk Scand J Med Sci Sports 2009 19 6 764 81 10.1111/j.1600-0838.2009.00992.x 19705997

[B8] Colbert LH Lanza E Ballard-Barbash R Adenomatous polyp recurrence and physical activity in the Polyp Prevention Trial (United States) Cancer Causes Control 2002 13 5 445 453 10.1023/A:1015736524447 12146849

[B9] Giovannucci E Ascherio A Rimm EB Colditz GA Stampfer MJ Willett WC Physical activity, obesity, and risk for colon cancer and adenoma in men Ann Intern Med 1995 122 5 327 334 7847643 10.7326/0003-4819-122-5-199503010-00002

[B10] Giovannucci E Colditz GA Stampfer MJ Willett WC Physical activity, obesity, and risk of colorectal adenoma in women (United States) Cancer Causes Control 1996 7 2 253 263 10.1007/BF00051301 8740738

[B11] Kono S Shinchi K Ikeda N Yanai F Imanishi K Physical activity, dietary habits and adenomatous polyps of the sigmoid colon: a study of self-defense officials in Japan J Clin Epidemiol 1991 44 11 1255 1261 10.1016/0895-4356(91)90158-6 1941019

[B12] Neugut AI Terry MB Hocking G Leisure and occupational physical activity and risk of colorectal adenomatous polyps Int J Cancer 1996 68 6 744 748 10.1002/(SICI)1097-0215(19961211)68:6<744::AID-IJC9>3.0.CO;2-3 8980177

[B13] Sandler RS Pritchard ML Bangdiwala SI Physical activity and the risk of colorectal adenomas Epidemiology 1995 6 6 602 606 10.1097/00001648-199511000-00007 8589091

[B14] Terry MB Neugut AI Bostick RM Risk factors for advanced colorectal adenomas: a pooled analysis Cancer Epidemiol Biomarkers Prev 2002 11 7 622 629 12101109

[B15] Dowse GK Zimmet PZ Gareeboo H Abdominal obesity and physical inactivity as risk factors for NIDDM and impaired glucose tolerance in Indian, Creole, and Chinese Mauritians Diabetes Care 1991 14 4 271 282 10.2337/diacare.14.4.271 2060430

[B16] Regensteiner JG Mayer EJ Shetterly SM Relationship between habitual physical activity and insulin levels among nondiabetic men and women. San Luis Valley Diabetes Study Diabetes Care 1991 14 11 1066 1074 10.2337/diacare.14.11.1066 1797488

[B17] Emmons KM McBride CM Puleo E Marcus BH Prevalence and predictors of multiple behavioral risk factors for colon cancer Prev Med 2005 40 5 527 534 10.1016/j.ypmed.2004.10.001 15749134

[B18] Rosenberg L Boggs D Wise LA Palmer JR Roltsch MH A Follow-up study of physical activty and incidence of colorectal polyps in African-American women Cancer Epidemiol Biomarkers Prev 2006 15 8 1438 1442 10.1158/1055-9965.EPI-06-0079 16896029

[B19] Chattar-Cora D Onime GD Valentine IS Cudjoe E Rivera L Colorectal cancer in a multi-ethnic urban group: its anatomical and age profile Int Surg 2000 85 2 137 142 11071331

[B20] Gonzalez EC Roetzheim RG Ferrante JM Campbell R Predictors of proximal vs. distal colorectal cancers Dis Colon Rectum 2001 44 2 251 258 10.1007/BF02234301 11227943

[B21] Theuer CP Taylor TH Brewster WR Campbell BS Becerra JC Anton-Culver H The topography of colorectal cancer varies by race/ethnicity and affects the utility of flexible sigmoidoscopy Am Surg 2001 67 12 1157 1161 11768820

[B22] Francois F Park J Bini EJ Colon pathology detected after a positive screening flexible sigmoidoscopy: a prospective study in an ethnically diverse cohort Am J Gastroenterol 2006 101 4 823 830 10.1111/j.1572-0241.2006.00433.x 16494591

[B23] Rex DK Johnson DA Lieberman DA Burt RW Sonnenberg A Colorectal cancer prevention 2000: screening recommendations of the American College of Gastroenterology. American College of Gastroenterology Am J Gastroenterol 2000 95 4 868 877 10763931 10.1111/j.1572-0241.2000.02059.x

[B24] Winawer S Fletcher R Rex D Colorectal cancer screening and surveillance: clinical guidelines and rationale-Update based on new evidence Gastroenterology 2003 124 2 544 560 10.1053/gast.2003.50044 12557158

[B25] Pechter U Ots M Mesikepp S Beneficial effects of water-based exercise in patients with chronic kidney disease Int J Rehabil Res 2003 26 2 153 156 10.1097/00004356-200306000-00013 12799612

[B26] Larsen IK Grotmol T Almendingen K Hoff G Lifestyle as a predictor for colonic neoplasia in asymptomatic individuals BMC Gastroenterol 2006 6 5 10.1186/1471-230X-6-5 16412216 PMC1374667

[B27] Wallace K Baron JA Karagas MR The association of physical activity and body mass index with the risk of large bowel polyps Cancer Epidemiol Biomarkers Prev 2005 14 9 2082 2086 10.1158/1055-9965.EPI-04-0757 16172213

[B28] Kim SE Shim KN Jung SA Yoo K Moon IH An association between obesity and the prevalence of colonic adenoma according to age and gender J Gastroenterol 2007 42 8 616 623 10.1007/s00535-007-2074-4 17701124

[B29] Morois S Mesrine S Josset M Clavel-Chapelon F Boutron-Ruault MC Anthropometric factors in adulthood and risk of colorectal adenomas: The French E3N-EPIC prospective cohort Am J Epidemiol 2010 172 10 1166 1180 10.1093/aje/kwq258 20858743

[B30] Siddiqui A Chang M Mahgoub A Sahdala HN Increase in Body Size Is Associated with an Increased Incidence of Advanced Adenomatous Colon Polyps in Male Veteran Patients Digestion 2011 83 4 288 290 10.1159/000322042 21282954

[B31] Wernli KJ Newcomb PA Wang Y Body size, IGF and growth hormone polymorphisms, and colorectal adenomas and hyperplastic polyps Growth Horm IGF Res 2010 20 4 305 309 10.1016/j.ghir.2010.04.001 20580999 PMC2918710

[B32] Nilsen TI Romundstad PR Petersen H Gunnell D Vatten LJ Recreational physical activity and cancer risk in subsites of the colon (the Nord-Trondelag Health Study) Cancer Epidemiol Biomarkers Prev 2008 17 1 183 188 10.1158/1055-9965.EPI-07-0746 18199723

[B33] Oettle GJ Effect of moderate exercise on bowel habit Gut 1991 32 8 941 944 10.1136/gut.32.8.941 1885077 PMC1378967

[B34] Levin B Lieberman DA McFarland B Smith RA Screening and surveillance for the early detection of colorectal cancer and adenomatous polyps, 2008: a joint guideline from the American Cancer Society, the US Multi-Society Task Force on Colorectal Cancer, and the American College of Radiology CA Cancer J Clin 2008 58 3 130 160 10.3322/CA.2007.0018 18322143

